# *Helobdella blinni* sp. n. (Hirudinida, Glossiphoniidae) a new species inhabiting Montezuma Well, Arizona, USA

**DOI:** 10.3897/zookeys.661.9728

**Published:** 2017-03-15

**Authors:** Rebecca K. Beresic-Perrins, Fredric R. Govedich, Kelsey Banister, Bonnie A. Bain, Devin Rose, Stephen M. Shuster

**Affiliations:** 1 Department of Biological Sciences, Northern Arizona University, 617 S. Beaver St., PO Box 5640 Flagstaff, AZ, 86011-5640; 2 Department of Biological Sciences, Southern Utah University, 351 W. University Blvd. Cedar City, UT, 84720

**Keywords:** Leech, Glossiphoniidae, *Helobdella blinni* sp. n., new species, Montezuma Well

## Abstract

A new leech species *Helobdella blinni* **sp. n.**, is described from Montezuma Well, an isolated travertine spring mound located in central Arizona, USA. In its native habitat, *Helobdella blinni* had been previously identified as *Helobdella stagnalis* (Linnaeus, 1758), which was later reclassified to *Helobdella modesta* (Verrill, 1872). Similar to the European *Helobdella stagnalis* and North American *Helobdella modesta*, *Helobdella blinni* has six pairs of testisacs, five pairs of smooth crop caecae, one lobed pair of posteriorly-directed crop caecae, one pair of eyes, a nuchal scute, and diffuse salivary glands. However, the pigmentation of this new species ranges from light to dark brown, unlike *Helobdella modesta* which tends to be light grey in color. Also, *Helobdella modesta* produces a clutch of 12-–35 pink eggs, whereas *Helobdella blinni* produces smaller clutches of white eggs (7–14, 0.5 ± 0.15 mm, N = 7) and consequently broods fewer young (1–14, 7 ± 3.3 mm, N = 97). *Helobdella blinni* are also able to breed year-round due to the constant warm water conditions in Montezuma Well. Their breeding season is not restricted by seasonal temperatures. These species are morphologically similar, however, comparing the COI mtDNA sequences of *Helobdella blinni* with sequences from nearby populations of *Helobdella modesta* and other *Helobdella* species from GenBank indicate that *Helobdella blinni* is genetically distinct from these other *Helobdella* populations.

## Introduction

**Figure 1.**
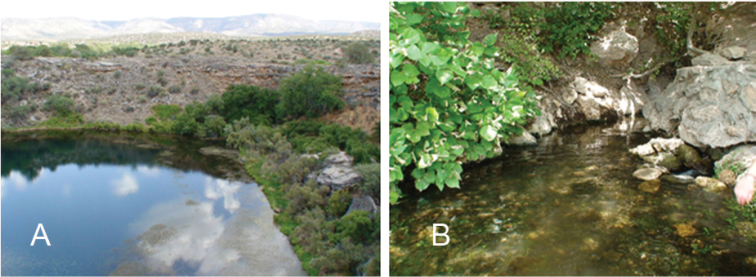
Location of *Helobdella blinni* sp. n. **A** The northeast side of Montezuma Well; and **B** The swallet where the leeches were collected.

Montezuma Well is a collapsed travertine spring mound located 72 km south of Flagstaff in the Verde Valley of Northern Arizona (34.6491°N,111.7522°W (DD)) (Fig. 1A). The age of Montezuma Well is estimated to be ~11,000 years (Wagner and Blinn 1987). This location is thermally constant year-round (19–24˚C) and is continuously replenished by two vents located at the well bottom. Montezuma Well is 0.76 ha in area and approximately 20 m deep. Most of the shoreline drops off immediately into open water, except at the northeast corner where water drains through a shallow region called the “swallet” and empties into Wet Beaver Creek which is located east of Montezuma Well (Fig. 1A–B). The water within Montezuma Well has unique water chemistry, containing high levels of arsenic (>100μg/L) and dissolved CO_2_ (>300mg/L) (Cole and Barry 1973).

Four leech species are known to inhabit Montezuma Well, including an endemic pelagic predator (Govedich et al. 1998), the erpobdellid *Motobdella montezuma* (Davies et al. 1985), and three other glossiphoniid species currently identified as *Helobdella papillata* (Moore, 1952), *Helobdella elongata* (Castle, 1900), and a species currently thought to be *Helobdella stagnalis* (Linnaeus, 1758), all of which inhabit the swallet (Fig. 1B). These Montezuma Well leech populations are thought to have been isolated from other leech populations for as long as 11,000 years (Wagner and Blinn 1987).

In support of this hypothesis, Beresic-Perrins’s (2010) description of brood size, parental behavior, and life history of the Montezuma Well population of *Helobdella stagnalis* suggests that this leech is distinct from other known populations of *Helobdella stagnalis*, a species originally described from Europe and which had until very recently been considered to be a widespread cosmopolitan leech species, inhabiting both Europe and North America. Siddall et al. (2005) addressed this problem by resurrecting the original species description for the North American leech, *Helobdella modesta* (Verrill, 1872) which had long been considered to be a synonym of the European *Helobdella stagnalis* (Moore 1898). The molecular analysis by Moser et al. (2011) provided confirmation for the resurrection of *Helobdella modesta* by Siddall et al. (2005). Even though the two species are morphologically indistinguishable (Verrill 1872, Moore 1898, Moore 1952), they differ genetically. Henceforth, we will refer to the North American *Helobdella stagnalis* as *Helobdella modesta*.

Here, we compare key traits, both morphological and molecular, among members of the Montezuma Well *Helobdella* sp. population, several other nearby populations of *Helobdella modesta*, and several other *Helobdella* species. Our molecular analysis includes the cytochrome *c* oxidase subunit I (COI) mitochondrial gene region to test the hypothesis that the Montezuma Well population of *Helobdella modesta* is a distinct species and warrants a new species description. This region is known to be sufficiently variable to reveal interspecific differences and unlikely to suggest differences due to elevated mutation rates (Apakupakul et al. 1999).

## Materials and methods

### Sampling

A total of 34 individuals of *Helobdella* sp. inhabiting Montezuma Well were collected from the underside of rocks in the swallet: five specimens were collected in June 2011 for molecular analysis and 29 were collected in June 2012 to assess morphological characteristics. For the molecular analysis, the leeches were preserved in 95% ethanol and others, for museum collections, were fixed with buffered formalin overnight and preserved in 70% ethanol. Additionally, a total of 10 specimens of Helobdella c. f. modesta from Rio de Flag ponds near the Rio de Flag Waste Water Facility outflow in Flagstaff, Arizona (35.18418°N, 111.63294°W (DD)) and Oak Creek, AZ near the Cave Springs campground (34.9961°N, 111.7394°W (DD)) were collected for molecular analyses. These specimens were also fixed in 95% ethanol.

### Morphological examination

We documented number of eyes and their placement, color pattern, presence of papillae, number of and structure of gastric caecae, body size, presence of nuchal scute, gonopore placement, egg size and number, and number of offspring using a Nikon binocular dissecting microscope. We then deposited the examined materials in the Invertebrate Zoology collection at the Smithsonian Institution, National Museum of Natural History (USNM).

### Molecular analysis

Whole DNA was extracted from the caudal suckers of the individual leeches using a Qiagen DNeasy Blood & Tissue Kit (Cat. No. 69504), with each sample incubated overnight in a water bath set at 54°C. Using Siddall and Borda’s (2002) PCR method, the mitochondrial gene region, cytochrome *c* oxidase subunit I (COI) was amplified. The primers were LCO1490 5’-GGTCAACAAATCATAAAGATATTGG-3’ and HCO2198 5’-TAAACTTCAGGGTGACCAAAAAATCA-3’ (Folmer et al. 1994). The PCR product was purified through the use of the QIAquick PCR Purification Protocol (Cat. No. 28104), checked for PCR product using gel electrophoresis, and sequenced with an ABI Prism 3730 sequencer (Applied Biosystems). We imported the seven “cleanest” sequences and 71 comparative sequences (Table 1) from previous studies (Siddall and Burreson 1998, Siddall and Borda 2002, Siddall and Budinoff 2005, Siddall et al. 2005, Williams and Burreson 2005, Bely and Weisblat 2006, Williams and Burreson 2006, Lai et al. 2009, Oceguera-Figueroa et al. 2010, Kutschera 2011, Moser et al. 2013) from GenBank (http://www.ncbi.nlm.nih.gov/genbank/) into MEGA7.0.18 (Kumar et al. 2016). We aligned the sequences automatically using MUSCLE (Edgar 2004) and then corrected the alignments by hand. We partitioned the data and performed the substitution model test by codon in Partitionfinder (Lanfear et al. 2012). The best substitution model test was General Time Reversal (GTR) +gamma which we used in our maximum-likelihood (ML) analysis (Lanave et al. 1984, Tavare 1986, Rodriguez et al. 1990). For ML analysis, we used RAxML v. 8 (Stamatakis 2014) and included 1,000 nonparametric bootstrap replicates. We used MrBayes for Bayesian inference analysis with ten million generations with a 25% burn-in and our support was assessed based on clade posterior probabilities (Ronquist and Huelsenbeck 2003). These analyses were conducted through CIPRES (Miller et al. 2010). We used PAUP* 4.0 (Swofford 2003) to construct parsimony phylogenies with 100 random additions. We performed the parsimony analysis twice, treating the deletions in the sequences as a 5^th^ state and then as missing data. We performed an uncorrected p-distance analysis to examine nucleotide differences between sequences with 1,000 replicates in MEGA7.0.18 (Kumar et al. 2016).

**Table 1.** *Helobdella* and outgroup taxa used for our molecular analysis.

Taxon	Locality	Reference
*Cystobranchus salmositicus*	Outgroup	Williams and Burreson 2006
*Ozobranchus margoi*	Outgroup	Siddall and Burreson 1998
*Gonimosobdella klemmi*	Outgroup	Williams and Burreson 2005
*Myzobdella lugubris*	Outgroup	Siddall and Burreson 1998
*Helobdella atli*	French Guiana	Oceguera-Figueroa et al. 2010
*Helobdella atli*	Uruguay	Oceguera-Figueroa et al. 2010
*Helobdella atli*	Mexico	Oceguera-Figueroa et al. 2010
*Helobdella blinni* sp. n.	Montezuma Well, AZ, USA	This study
*Helobdella blinni* sp. n.	Montezuma Well, AZ, USA	This study
*Helobdella blinni* sp. n.	Montezuma Well, AZ, USA	This study
*Helobdella bolivianita*	Bolivia	Siddall and Borda 2002
*Helobdella bowermani*	Oregon, USA	Moser et al. 2013
*Helobdella bowermani*	Oregon, USA	Moser et al. 2013
*Helobdella bowermani*	Oregon, USA	Moser et al. 2013
*Helobdella californica*	California, USA	Kutschera 2011
*Helobdella* “*elongata*”	Mexico	Oceguera-Figueroa et al. 2010
*Helobdella europaea*	Taiwan	Lai et al. 2009
*Helobdella europaea*	Taiwan	Lai et al. 2009
*Helobdella europaea*	Taiwan	Lai et al. 2009
*Helobdella europaea*	Taiwan	Lai et al. 2009
*Helobdella europaea*	South Africa	Siddall and Budinoff 2005
*Helobdella lineata*	Michigan, USA	Siddall and Borda 2002
*Helobdella fusca*	Michigan, USA	Siddall and Borda 2002
*Helobdella melananus*	Taiwan	Lai et al. 2009
*Helobdella melananus*	Taiwan	Lai et al. 2009
*Helobdella melananus*	Taiwan	Lai et al. 2009
*Helobdella michaelseni*	Chile	Siddall and Borda 2002
*Helobdella modesta*	Columbus, Ohio, USA	Siddall and Borda 2002
*Helobdella modesta*	Washington, USA	Oceguera-Figueroa et al. 2010
*Helobdella modesta*	Washington, USA	Oceguera-Figueroa et al. 2010
Helobdella c. f. modesta	Rio de Flag, Flagstaff, AZ, USA	This study
Helobdella c. f. modesta	Rio de Flag, Flagstaff, AZ, USA	This study
Helobdella c. f. modesta	Oak Creek, AZ, USA	This study
Helobdella c. f. modesta	Oak Creek, AZ, USA	This study
*Helobdella nununununojensis*	Bolivia	Siddall and Borda 2002
*Helobdella nununununojensis*	Bolivia	Siddall and Borda 2002
*Helobdella octatestisaca*	Taiwan	Lai et al. 2009
*Helobdella octatestisaca*	Taiwan	Lai et al. 2009
*Helobdella octatestisaca*	Taiwan	Lai et al. 2009
*Helobdella octatestisaca*	Taiwan	Lai et al. 2009
*Helobdella octatestisaca*	Taiwan	Lai et al. 2009
*Helobdella octatestisaca*	Taiwan	Lai et al. 2009
*Helobdella octatestisaca*	Taiwan	Lai et al. 2009
*Helobdella octatestisaca*	South Africa	Oceguera-Figueroa et al. 2010
*Helobdella octatestisaca*	Mexico	Oceguera-Figueroa et al. 2010
*Helobdella octatestisaca*	Mexico	Oceguera-Figueroa et al. 2010
*Helobdella octatestisaca*	Mexico	Oceguera-Figueroa et al. 2010
*Helobdella octatestisaca*	Mexico	Oceguera-Figueroa et al. 2010
*Helobdella octatestisaca*	Mexico	Oceguera-Figueroa et al. 2010
*Helobdella papillata*	Michigan, USA	Siddall and Borda 2002
*Helobdella papillata*	Virginia, USA	Siddall and Borda 2002
*Helobdella papillornata*	Australia	Siddall and Borda 2002
*Helobdella paranensis*	Uruguay	Siddall and Borda 2002
*Helobdella pichipanan*	Bolivia	Siddall et al. 2005
*Helobdella* “*robusta*” TXAU1	Texas, USA	Bely and Weisblat 2006
*Helobdella* “*robusta*”	California, USA	Bely and Weisblat 2006
*Helobdella* “*robusta*” CASA 1	California, USA	Bely and Weisblat 2006
*Helobdella* “*robusta*” NYTA	New York, USA	Bely and Weisblat 2006
*Helobdella simplex*	Argentina	Moser et al. 2006
*Helobdella simplex*	Argentina	Moser et al. 2006
*Helobdella simplex*	Argentina	Moser et al. 2006
*Helobdella socimulcensis*	Mexico	Oceguera-Figueroa et al. 2010
*Helobdella socimulcensis*	Mexico	Oceguera-Figueroa et al. 2010
*Helobdella socimulcensis*	Mexico	Oceguera-Figueroa et al. 2010
*Helobdella socimulcensis*	Mexico	Oceguera-Figueroa et al. 2010
*Helobdella socimulcensis*	Mexico	Oceguera-Figueroa et al. 2010
*Helobdella socimulcensis*	Mexico	Oceguera-Figueroa et al. 2010
*Helobdella socimulcensis*	Mexico	Oceguera-Figueroa et al. 2010
*Helobdella* sp. Xochimilco	Mexico	Oceguera-Figueroa et al. 2010
*Helobdella sorojchi*	Bolivia	Siddall and Borda 2002
*Helobdella sorojchi*	Bolivia	Siddall and Borda 2002
*Helobdella stagnalis*	United Kingdom	Siddall and Borda 2002
*Helobdella* “*stagnalis*”	Mexico	Oceguera-Figueroa et al. 2010
*Helobdella* “*stagnalis*”	Mexico	Oceguera-Figueroa et al. 2010
*Helobdella transversa*	Michigan, USA	Siddall and Borda 2002
*Helobdella triserialis*	Bolivia	Siddall and Borda 2002
*Helobdella triserialis*	California, USA	Bely and Weisblat 2006
*Helobdella virginiae*	Mexico	Oceguera-Figueroa et al. 2010

## Results

### Family Glossiphoniidae Vaillant, 1890

#### Genus *Helobdella* Blanchard, 1896

##### Helobdella blinni sp. n.

Taxon classificationAnimaliaRhynchobdellidaGlossiphoniidae

http://zoobank.org/B1B3D234-BC3F-4126-BF25-52DA00BA7EB9

Figs 2 , 3 , 4

###### Type materials.

**Holotype.** USNM 1186106 (Table 2).

**Table 2.** Holotype and paratype collection data and voucher numbers.

Family	Species	Collection data	Voucher #
Glossiphoniidae	*Helobdella blinni* sp. n.	USA: AZ: Yavapai Co., Montezuma Well 34.6491°N, 111.7522°W (DD), 10.VI.2010, aquatic system, under rocks, RK Beresic-Perrins, Holotype (USNM)	1186106
Glossiphoniidae	*Helobdella blinni* sp. n.	(14 specimens) USA: AZ Yavapai Co., Montezuma Well 34.6491°N, 111.7522°W (DD), 10.VI.2010, aquatic system, under rocks, RK Beresic-Perrins, Paratypes (USNM)	1186107
			1186108
			1186109
			1186110
			1186111
			1186112
			1186113
			1186114
			1186115
			1186116
			1186117
			1186118
			1186119
			1186120

###### Additional materials.

**Paratypes**. (14 specimens) (USNM 1186107, 1186108, 1186109, 1186110, 1186111, 1186112, 1186113, 1186114, 1186115, 1186116, 1186117, 1186118, 1186119, 1186120) (Table 2)

###### Type locality.

USA, Arizona: Yavapai County, Montezuma Well (34.6491°N, 111.7522°W (DD)), aquatic system, under rocks, 10 June 2012, R.K. Beresic-Perrins.

###### Etymology.

We have named this new species, *Helobdella blinni* in honor of Dr. Dean W. Blinn for his dedication to natural history research at Montezuma Well. For over 20 years at Northern Arizona University, Dr. Blinn studied a wide range of organisms and their interactions at Montezuma Well including predator-prey interactions between *Motobdella montezuma* and the endemic amphipod, *Hyalella montezuma* Cole & Watkins, 1977.

###### Description.

**Figure 2.**
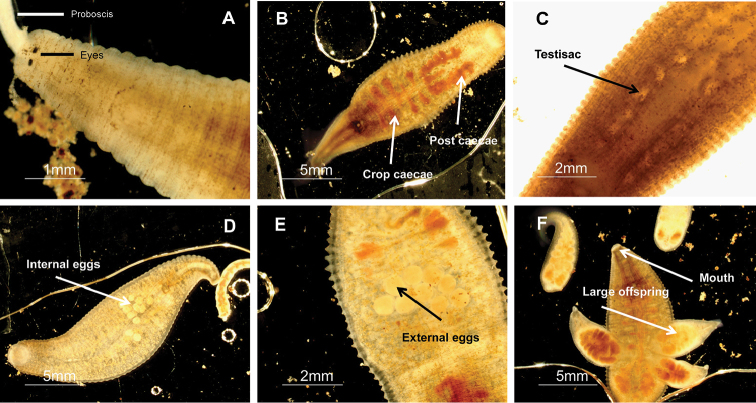
Internal and external morphology of *Helobdella blinni* sp. n. **A** dorsal view of the eyes and extended proboscis **B** crop and post caecae **C** testisacs **D** ventral view of internal eggs which have not been oviposited yet **E** ventral view of white eggs that have been oviposited **F** ventral view of attached and detached offspring.

**Figure 3.**
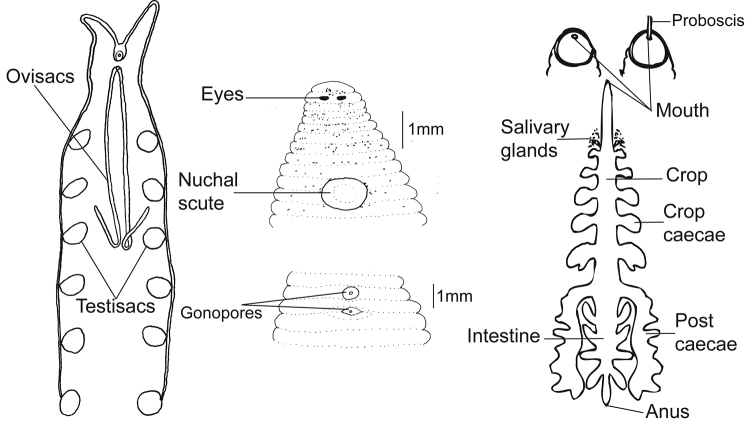
Diagram of the external and internal morphology of *Helobdella blinni* sp. n. (drawn by Rebecca Beresic-Perrins and Fredric Govedich).

**Figure 4.**
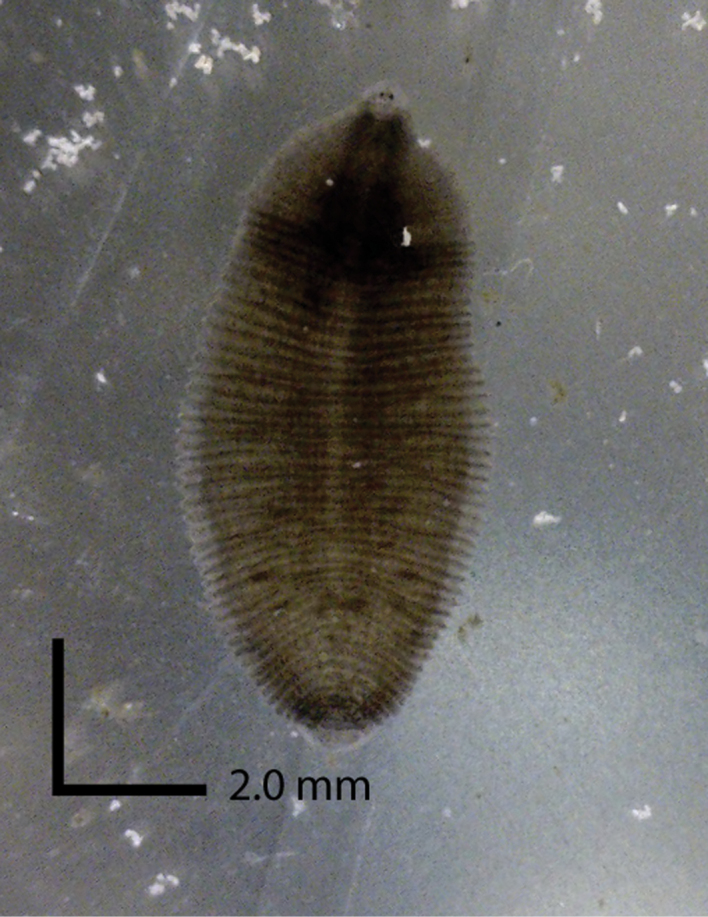
Typical pigmentation of *Helobdella blinni* sp. n.

**External morphology.** Length of specimens 11 to 22 mm (mean + SE 16.6 + 3.2 N=24) and width 3 to 8 mm (5.7 + 1.1 N=28) (Table 3, Figs 2, 3). Individual color ranges from translucent with brown spots to dark brown (Fig. 4). No dorsal papillae; one pair of eyes located at somite II (0.07 + 0.02 mm diameter, N = 11), distance between eyes 0.1 to 0.03 mm apart (N = 13). A scallop-shaped nuchal scute is present on the dorsal side, length 0.293 to 0.432 mm (0.335 + 0.05 N=9) and width 0.27 to 0.386 mm (0.32 + 0.04 N=9). One annulus separates the female and male gonopores. The caudal sucker diameter averages 1.6 + 0.3 mm (N = 27). The eggs (diameter 0.5 + 0.15 mm, N = 28) are laid on the ventral side of the parent in soft-walled transparent cocoons (7–11 eggs per cocoon, N = 7). The mouth is located subterminally in the oral sucker (Figs 2, 3).

**Internal morphology.** Average oral sucker diameter is 0.7 + 0.19 mm (N = 15), proboscis length is 3.5 + 1.1 mm (N = 17) (Table 3). Diffuse salivary glands are located near the anterior of the first pair of crop caecae. There are five pairs of smooth crop caecae and one lobed pair of posteriorly directed post caecae. Six pairs of compact testisacs are located in between each of the crop caecae. The intestine contains four pairs of caecae, with the first two pairs anteriorly directed and the other two pairs posteriorly directed. The intestine leads into an unraised anus located two annuli from the caudal sucker (Figure 3).

**Development and growth.** This species breeds year-round with peaks in spring and fall. Our specimens had an average of 7 to 11 white eggs (diameter 0.5 + 0.15 mm, N = 7) fixed to their ventral surface. Laboratory collections (2007–10) of *Helobdella blinni* documented the eggs hatching 1 to 2 weeks after ovipositing (Beresic-Perrins 2010). Once hatched, the young attach to the ventral surface of the parent, allowing the parent to hunt for food and feed the young, occasionally feeding along with them. Prey consists of oligochaetes and other invertebrates. The average number of young per adult is 7 + 3.3 (N = 97) ranging from 1 to 14 offspring. The young remain attached to the parent for an additional four to five weeks after hatching. Once the juveniles leave the parent, they tend to aggregate together on rocks (Beresic-Perrins 2010).

**Table 3.** Morphological measurements of *Helobdella blinni* sp. n.

Trait	Ave	SE	Min	Max	N
body length relaxed (mm)	16.6	3.18	11.3	22.5	24
body width relaxed (mm)	5.7	1.15	3.1	8.0	28
caudal diameter (mm)	1.7	0.3	1.0	2.3	27
egg diameter (mm)	0.5	0.15	0.2	0.7	28
gonopore separation (mm)	0.1	0.08	0.1	0.3	13
nuchal scute length (mm)	0.335	0.05	0.284	0.432	9
nuchal scute width (mm)	0.32	0.04	0.27	0.386	9
proboscis length (mm)	3.5	1.10	2.0	6.2	17
oral sucker diameter (mm)	0.7	0.19	0.4	1.0	15
progeny length (mm)	3.6	1.68	1.6	6.6	18
progeny width (mm)	1.5	0.8	0.7	2.9	18
# eggs	10.0	2.73	7.0	16.0	7
# progeny	7.2	3.35	1.0	14.0	97
eye diameter (mm)	0.1	0.02	0.0	0.1	11
eye distance (mm)	0.1	0.04	0.0	0.2	13

##### Molecular analysis

**Figure 5.**
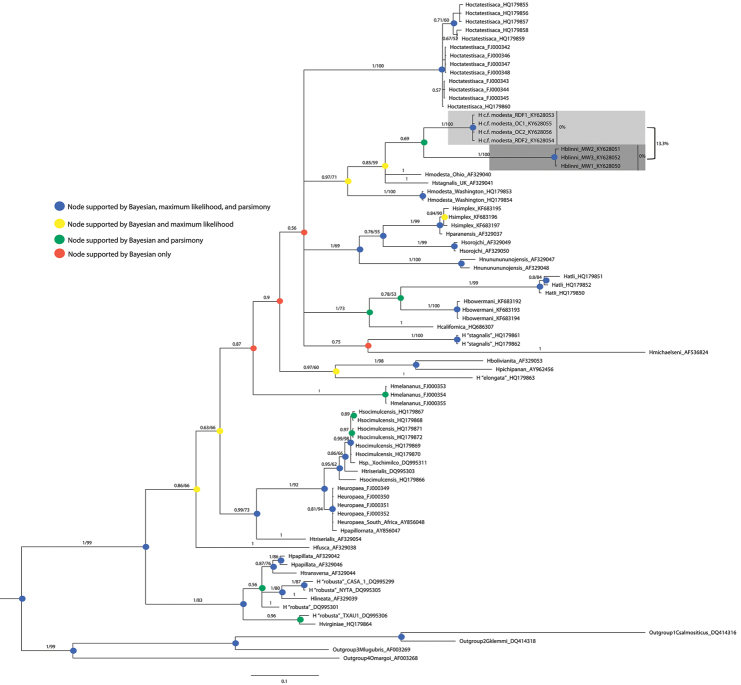
Bayesian Inference phylogenetic tree with 25% burn-in and support was assessed based on clade posterior probabilities tree. We included COI sequences from 31 species of *Helobdella* (family Glossiphoniidae). The Arizona populations are from Oak Creek (OC), Rio de Flag (RDF), and Montezuma Well (MW). Our outgroup included *Cystobranchus salmositicus* (Meyer, 1946), *Gonimosobdella klemmi* (Williams & Burreson, 2005), *Myzobdella lugubris* (Leidy, 1851), and *Ozobranchus margoi* (Davies, 1978). The shaded branches are the Arizona sample sequences. Branch labels include the Bayesian / ML probability. The blue nodes are supported by Bayesian Inference, Maximum-Likelihood, and parsimony analyses. The yellow nodes are supported by Bayesian Inference and Maximum-Likelihood analyses. The green nodes are supported by Bayesian Inference and parsimony analyses. The red nodes are supported by Bayesian Inference analysis only.

A Bayesian inference phylogenetic tree of the COI sequence data is presented in Figure 5. We include the posterior probabilities and maximum-likelihood branch supports >50. The Arizona populations of Helobdella c. f. modesta formed a sister clade to *Helobdella blinni* sp. n., supported by both the Bayesian and parsimony analyses. The results of the uncorrected p-distance analysis revealed a difference of 13.3% (233 nucleotides included) between the two groups (Table 6). The two groups form a larger clade with *Helobdella modesta* (Ohio), *Helobdella stagnalis* (UK), and *Helobdella modesta* (Washington) which is supported by both Bayesian inference and maximum-likelihood. *Helobdella blinni* differed from *Helobdella modesta* (Ohio) by 13.7%, *Helobdella stagnalis* (UK) by 16.3%, and *Helobdella modesta* (Washington) by 16.3% (Table 6).

When we aligned all 78 sequences, there were four, ten-codon deletions within all of the Arizona sequences and *Helobdella atli* (Oceguera-Figueroa and León-Regagnon 2005, Oceguera-Figueroa et al. 2010). When we performed the parsimony analysis, we included deletions as a 5^th^ state in our first analysis and in our second, we treated the deletions as missing data. In the resulting 5^th^ state tree, the two Arizona species remained sister taxa (100% support), but included in the clade was *Helobdella atli* (100% and 58% support). The missing data tree placed *Helobdella blinni* ancestral to *Helobdella modesta* (Washington), *Helobdella modesta* (Ohio), *Helobdella stagnalis* (UK), and Helobdella c. f. modesta with 100% branch support (Fig. 5).

**Table 4.** Morphological comparison of *Helobdella* species.

Traits	*Helobdella blinni* sp. n.	*Helobdella modesta*	*Helobdella californica*	*Helobdella papillornata*	*Helobdella temiscoensis*	*Helobdella atli*	*Helobdella bowermani*	*Helobdella octatestisaca*
	(current paper)	(Kutschera 1988: Sawyer 1986)	(Kutschera 1988; 2011)	(Govedich and Davies 1998)	(Salas-Montiel et al. 2014)	(Oceguera-Figueroa and León-Regagnon 2005)	(Moser et al. 2013)	(Lai et al. 2009)
crop caecae	5 pairs, smooth	5 pairs, smooth	6 pairs, lobed	5–6 pairs, lobed	4 pairs	6 pairs	5 pairs, smooth	5 pairs
post caecae	1 pair	1 pair	none	none	1 pair	none	1 pair	1 pair
eyes	1 pair	1 pair	1 pair	1 pair)	1 pair	1 pair	1 pair	1 pair
distance between eyes	0.1 mm	?	?	0.06 mm	?	?	?	?
nuchal scute	yes	yes	yes	no	yes	yes	yes	yes
pairs of testisacs	6 pairs	6 pairs	6 pairs	5 pairs	6 pairs	6 pairs	6 pairs	4 pair
salivary glands	diffuse	diffuse	?	diffuse	diffuse	?	diffuse	diffuse
proboscis length	3.5mm	?	0.7mm	2mm	?	?	?	?
color	transparent with spots to dark brown	transparent to light grey	dark grey	transparent with stripes and papillae	pale brown, blackish - on posterior and mid-body	white-yellowish	pale yellow/buff, papillae present	brown, pale, gray, and pink
body length	11–22 mm	8–12 mm	10–18 mm	15–40 mm	7.9–13.6 mm	7.5 mm	5.2–9.7 mm	9–14 mm
feeding	small invertebrates	small invertebrates	small invertebrates	small invertebrates	?	?	?	small invertebrates
brooding period	6–7 weeks	6–7 weeks	3–4 weeks	4–6 weeks	?	?	?	?
egg color	white	pink	pink	pink	?	?	?	?
egg diameter	0.5 mm	?	0.5 mm	0.2 mm	?	?	?	?
# eggs	7–14	12–35	8–56	20–50	?	?	?	?

**Table 5.** Differences in brooding season and size between *Helobdella blinni* sp. n., *Helobdella stagnalis*, and *Helobdella* c.f. *Helobdella*.

Location	Brooding Season	Average # of offspring	Author
*Helobdella blinni* sp. n. Montezuma Well, AZ	Year-round	1–14	Beresic-Perrins (2010)
*Helobdella modesta* Utah Lake, UT	Late spring through summer	12.6–17.4	Tillman and Barnes (1973)
*Helobdella modesta* Lake Washington, WA	Spring and Summer	14.5	Thut (1969)
*Helobdella modesta* Marion Lake, BC, CA	Spring and Summer	17.2–19.7	Davies and Reynoldson (1976)
*Helobdella modesta* Newsome Pond, AB, CA	Late spring through summer	21.3	
*Helobdella modesta* Cambridge, MA	Spring	31	Castle (1900)
*Helobdella modesta* Michigan	Late spring through summer	35.3	Sawyer (1972)
*Helobdella stagnalis* Iceland	Late spring through summer	No data	Bruun (1938)
*Helobdella stagnalis* River Ely, South Wales	Late spring through summer	No data	Murphy and Learner (1982)
*Helobdella stagnalis* Whiteknights Lake, UK	Late spring through summer	13–17	Mann (1957)
*Helobdella stagnalis* Eglwys Nunydd, UK	Late spring through summer	14	Learner and Potter (1974)
*Helobdella stagnalis* Denmark	Late spring through summer	20	Bennike (1943)

**Table 6.** Uncorrected p-distance pairwise analysis.

**Species**	**Distance - *Helobdella blinni***	**Distance - Helobdella c. f. modesta**
*Helobdella atli*	14.1–15%	16.7%
*Helobdella bolivianita*	18.5%	19.3%
*Helobdella bowermani*	15.9%	15.5%
*Helobdella blinni*	0.0%	13.3%
*Helobdella californica*	16.7%	17.6%
Helobdella c. f. modesta	13.3%	0.0%
*Helobdella elongata*	18.0%	19.3%
*Helobdella europaea*	15.5%	16.3%
*Helobdella fusca*	19.3%	20.6%
*Helobdella lineata*	16.3%	14.6%
*Helobdella melananus*	16.3%	17.2%
*Helobdella michaelseni*	23.2%	20.6%
*Helobdella modesta* OH	13.7%	8.6%
*Helobdella modesta* WA	16.3%	14.6%
*Helobdella nununununojensis*	17.5–19%	17.5–19.7%
*Helobdella octatestisaca*	19.7%	16.3%
*Helobdella papillata*	17.6%	15.8–16.3%
*Helobdella papillornata*	15.9%	16.7%
*Helobdella paranensis*	16.3%	13.7%
*Helobdella pichipanan*	17.2%	19.3%
*Helobdella robusta*	17.6%	14.6%
Helobdella aff robusta CASA	18.9%	17.2%
Helobdella aff robusta NYTA	17.6%	15.9%
Helobdella aff robusta TXAU1	17.2%	18.0%
*Helobdella simplex*	16.3%	13.7–14.2%
*Helobdella socimulcensis*	15.9%	16.7–17.2%
*Helobdella sorojchi*	18–18.5%	17.6%
*Helobdella* sp. Xochimilco	15.5%	17.2%
*Helobdella stagnalis*	20.6%	17.2%
*Helobdella stagnalis UK*	16.3%	11.6%
*Helobdella transversa*	16.3%	15.0%
*Helobdella triserialis*	15.9%	16.7–18.5%
*Helobdella virginiae*	16.3%	16.7%
Outgroup1 *Cystobranchus salmositicus*	24.9%	23.2%
Outgroup2 *Gonimosobdella klemmi*	21.5%	21.5%
Outgroup3 *Myzobdella lugubris*	18.0%	19.3%
Outgroup4 *Ozobranchus margoi*	23.2%	20.2%

## Discussion

*Helobdella blinni* sp. n. has morphological and life-history traits similar to other *Helobdella* species, including possession of a nuchal scute, diffuse salivary glands, six pairs of testisacs, and extended parental care for the young (6–7 weeks; Tables 4, 5). *Helobdella blinni*, *Helobdella bowermani* (Moser et al. 2013), *Helobdella octatestisaca* (Lai et al. 2009), and Helobdella c. f. modesta each have five pairs of smooth crop caecae as opposed to six pairs of lobed crop caecae in *Helobdella californica* (Kutschera 2011) and *Helobdella papillornata* (Govedich and Davies 1998). *Helobdella blinni* and *Helobdella temiscoensis* (Salas-Montiel et al. 2014) share pigmentation characteristics, but they differ internally. *Helobdella temiscoensis* has only four pairs of crop caecae and one descending post caecae as opposed to five pairs and one descending post caecae in *Helobdella blinni*. *Helobdella modesta*, *Helobdella californica*, *Helobdella atli*, *Helobdella bowermani*, and *Helobdella octatestisaca* do not resemble the pigmentation of *Helobdella blinni*, running the spectrum from grey to pink. Additionally, they have a descending pair of post caecae, whereas *Helobdella atli*, *Helobdella californica*, and *Helobdella papillornata* do not. *Helobdella blinni*, Helobdella c. f. modesta, *Helobdella californica*, *Helobdella temiscoensis*, *Helobdella atli*, and *Helobdella bowermani* possess six pairs of testisacs, whereas *Helobdella papillornata* has five pairs and *Helobdella octatestisaca* has four pairs. *Helobdella blinni* also has a larger proboscis than the other *Helobdella* species (mean + SE, *Helobdella blinni* 3.5 mm + 1.1, N=17, *Helobdella californica* mean = 0.7 mm, *Helobdella papillornata* mean= 2 mm). Furthermore, breeding periods also differ between *Helobdella blinni* and the other *Helobdella* species (Tables 4, 5).

*Helobdella blinni*, unlike the other *Helobdella* species discussed here, breeds year-round, living in the thermally stable environment of Montezuma Well, with constant (19–24˚C) year-round temperatures (Table 5). Monthly samples have individuals carrying cocoons every month of the year, with peak seasons in the spring and fall, a situation quite different than that for other *Helobdella* species, which have seasonally-constrained reproductive cycles, with egg-laying and brooding beginning in the spring and ending in the fall every year (Table 5). In addition to breeding year-round, *Helobdella blinni* produces smaller broods (7–14 young) when compared to *Helobdella modesta* and *Helobdella stagnalis* (12–35 young) (Tables 4, 5) and has white eggs, unlike the characteristically pink eggs of *Helobdella modesta*, *Helobdella californica*, and *Helobdella papillornata* (Table 4). The external pigmentation of *Helobdella blinni* also tends to be dark brown, whereas most other *Helobdella* species are grey/brown in color (Fig. 4). *Helobdella blinni* are slightly longer (body length 11–22 mm, 16.6 + 3.2, N=24) than Helobdella c. f. modesta (8–12 mm) and *Helobdella californica* (10–18 mm), but slightly shorter in length than *Helobdella papillornata* (15–40 mm) (Table 4).

The results from our molecular analysis show *Helobdella blinni* to be genetically distinct from other *Helobdella* species, both from the same region (Rio de Flag and Oak Creek, Arizona populations) and from Europe (UK sample). The sequences yielded a 13.3%–17.4% genetic difference between *Helobdella blinni* and both the Arizona Helobdella c. f. modesta, *Helobdella modesta* (Ohio and Washington), and the United Kingdom *Helobdella stagnalis* populations (Table 6). The three Arizona populations belong to their own separate clade, but are closely related to *Helobdella stagnalis* (UK) and the *Helobdella modesta*. *Helobdella atli*, *Helobdella bowermani*, and *Helobdella californica* are located on separate branch tips, but they comprise what Oceguera-Figueroa et al. (2010) designated as the “*stagnalis*” series (Fig. 5).

Based on morphological, life-history, and molecular differences, we propose the *Helobdella* sp. leeches found at Montezuma Well should be considered a new species, likely the result of allopatric isolation. This concept supports our hypothesis that the leech species inhabiting Montezuma Well may have become isolated from other populations as far back as 11,000 years ago (Wagner and Blinn 1987). *Helobdella blinni* sp. n. can be considered a distinct species found in Montezuma Well *and* may also turn out to be endemic to the area. Further sampling and analyses are needed in order to verify endemism.

Although currently classified as Helobdella c. f. modesta, the Arizona populations from the Rio de Flag and Oak Creek may be an additional undescribed species based on our molecular analysis. Our next step is to investigate these populations more closely, comparing them to other local populations, including White Horse Lake and J.D. Dam Lake, AZ which also contain *Helobdella modesta*.

## Supplementary Material

XML Treatment for Helobdella blinni
